# Family and neurosurgery - a balancing act between vocation and responsibility

**DOI:** 10.1016/j.bas.2026.107489

**Published:** 2026-10-23

**Authors:** Xenia Hautmann, Esther Neufeld, Nadine Lilla, Felix Behling, Dorothee Mielke, Peter Baumgarten

**Affiliations:** a Neurosurgery, Faculty of Medicine, University of Augsburg, Augsburg, Germany; b Neurology, Faculty of Medicine, University of Augsburg, Augsburg, Germany

**Keywords:** Family, Caring, Neurosurgery, Work-life balance, Parenthood

## Abstract

**Introduction:**

Reconciling a career in neurosurgery with caregiving responsibilities can present major challenges. While recent scientific work has increasingly focused on the disadvantages faced by women, less attention has been paid to the experiences of men who engage in caregiving in an increasing proportion. The potential tension between the role of a man taking over with caring and that of a neurosurgeon remains largely unexplored.

**Research question:**

The aim of our survey is to shed more light on the perceived disadvantages faced by neurosurgeons of both genders in balancing childcare with career advancement.

**Material and methods:**

We conducted a Germany-wide, 33-question online survey focusing on discrimination and burden due to “caring” in a neurosurgical career.

**Results:**

In total, 272 individuals responded to the survey. The male to female ratio was 173/98 (p < 0.001). Inappropriate comments on caring were perceived by 49.78% of respondents, with women being significantly more frequently affected (67.0% vs 39.4%, p < 0.001). During job interviews, 28.63% were asked about family planning with women being affected more often (40.9% vs. 21.4%, p < 0.001). On a satisfaction scale of 0-100 (0 = negative, 100 = positive), experienced disadvantages due to caring were rated at 48.1, management flexibility at 52.4, team flexibility at 61.2, and actual working conditions at 31.4.

**Discussion and conclusion:**

According to our data, a significant proportion of men are also affected by discrimination due to “caring,” even though women still appear to be affected to a greater extent. Working conditions are perceived as the worst factor for balancing caregiving and work.

## Highlights

• On a scale from 0 (negative) to 100 (positive) working conditions were rated at 31.4
• The question of family planning in job interviews was dependent on age for men, p = 0.011
• 72% stated, that a reduction in working hours led to conflicts in their team.

## 1 Introduction

Neurosurgery is among the most challenging medical specialties, characterized by a high workload, long working hours and great responsibility. A survey showed that nearly half of the resident physicians surveyed in Greece meet the criteria for burnout (Msaouel et al., 2010). There is growing awareness that reconciling a career in surgery with caregiving responsibilities can present major challenges for both genders. A survey among surgeons revealed, that – independent of their gender – only 32% saw their specialty as family-friendly. 18% stated, that the support they received for paternity/maternity leave was poor to very poor (Lambert et al., 2017). In a Europe-wide study, nearly 80% of the neurosurgeons surveyed said they wanted more workable parental leave policies for young parents (Lambrianou et al., 2022). In a survey among medical personnel supervisors, parenthood was seen as rather positive. However, 17% of employees in leadership positions stated, that they lost their leading status after paternity/maternity leave (Engelmann et al., 2015). While recent scientific work has increasingly focused on the disadvantages faced by women, less attention has been paid to the experiences of men who actively engage in caregiving. A Europe-wide survey showed that 72% of women worry about the negative impact that their wish to have children might have on their career, which is emphasized by a firm comprehension shared by 86% of respondents that family planning is rather ascribed to women (Wolfert et al., 2019). According to the Federal Statistical Office of Germany, in 2024 among all parents approximately 25% of women took maternal leave because they had children under the age of six, compared to only 1.8% of men ((Destatis and) SB, 2024). Although this indicates that men still take paternal leave far less frequently than women, it also represents nearly a doubling of the proportion of fathers taking leave since 2012 ((Destatis and) SB, 2024). A review of parental leave policies worldwide revealed significant variation, with substantially shorter periods of paid leave allowed for fathers in almost all countries (de Souza et al., 2022). This underscores the need to examine the role of men in childcare and the associated career conflicts to promote genuine gender equality. To date, no data on this topic have been published in the field of neurosurgery. A survey among orthopedic surgery residents showed that both genders delay their parenthood due to residency (Reid et al., 2021) – emphasizing the impact their career has on their family life. The need to evaluate this issue from the male perspective is underscored by the proportion of men who participated in the survey – 76.2% of respondents were male (Reid et al., 2021). On the other hand, it appears that gender-specific reasons and role models may be responsible for the choice of medical specialty. A study from North America suggests that unclear regulations regarding parental leave influence the choice of medical specialty (Jakubowski et al., 2023). Furthermore, among those who chose a surgical specialty, the proportion of women with children was lower than that of men (Au et al., 2021).

This study aims to investigate the extent to which childcare responsibilities affect neurosurgeons in Germany – both female and male. Furthermore, it may reveal factors that positively or negatively influence neurosurgeons’ choice for childcare in expense of their neurosurgical career.

## 2 Methods

### 2.1 Survey development

The questionnaire was developed specifically for this study by a diverse team of physicians with different genders, career stages, leadership roles, and family circumstances. Item selection was informed by personal and professional experiences and discussions with colleagues. The questionnaire was pretested with 15 residents and senior physicians at our institution, and unclear or redundant items were revised or removed. No formal psychometric validation was performed. The questionnaire included demographics, working hours, family and childcare responsibilities, partner employment, the experienced discrepancy between reality and expectation for childcare and working days as well as experiences of disadvantages related to family planning. Institutional affiliation was deliberately not collected following discussion within the research team and consideration of the ethical implications, as combining this information with gender and professional position could increase the risk of re-identification in rare respondent subgroups. The complete questionnaire is displayed in the appendix. Ethical approval was obtained by the ethics committee of the University Hospital Munich (No. 25-0942-KB). This study was performed in accordance with the 1964 Declaration of Helsinki and its later amendments.

### 2.2 Survey distribution

A nationwide, anonymous 33-item online survey (SurveyMonkey®) was distributed among all members of the German Society of Neurosurgery between 01/2025 and 06/2025. Participation was voluntary. All participants gave their informed consent prior to participation in the survey.

### 2.3 Statistics

Statistical analysis was performed using SPSS (IBM Version 30.0). We tested for normality using Shapiro-Wilk-Test. Continuous variables were analyzed using t-tests and Mann-Whitney-U test, categorical variables were analyzed with the chi-square test. Statistical significance was defined as p < 0.05. For descriptive analyses, all available responses were included, and the number of valid observations therefore varied across questionnaire items (Table 1). For questions that could only be answered meaningfully if a previous question was answered with “yes,” the response indicating missing data applies only to the corresponding subgroup. Participants with incomplete questionnaires were not excluded. For inferential analyses examining associations between multiple variables, participants with missing values on any variable required for the respective analysis were excluded from that analysis. No imputation was performed.

**Table 1:** Missing answers per question.

Question	N	
	valid	missing
gender	271	2
age	272	1
Are you board certified?	272	1
year of training	273	0
position	260	13
hours of teaching students per week	257	16
additional responsibilities at the hospital	260	13

**Part-time work**		
**Are you working full-time?** → n = 42 answered with no	260	13
To what percentage of your original weekly hours have you reduced your hours?	24	18
Did the reduction in working hours make your personal life easier?	25	17

What is your current weekly work schedule according to your employment contract, excluding any opt-out provisions?	273	0
How many hours do you estimate you actually work each week?	249	24
Do you plan to work part-time in the future?	259	14
Do you feel that a future reduction in working hours would have negative consequences in your team?	73	200
Do you feel that a reduction in working hours would result in your supervisor treating you unfavorably?	73	200
Have you ever reduced your working hours before?	257	16

**Relationship**		
**Are you in a committed relationship?** → n = 220 answered with yes	251	22
Is your partner employed?	220	0
Does your partner work full-time or part-time?	220	0
Does your partner work in the healthcare field?	219	1

**Children**		
**Do you have children?** → n = 180 answered with yes	251	22
How many children do you have?	165	15
How are the children being cared for?	161	19
Who, if anyone, helps you with childcare?	42	138
Have you ever felt disadvantaged because of your caregiving responsibilities?	161	19
How often have you felt disadvantaged because of child care responsibilities?	162	18
Do you feel that your supervisor is flexible when it comes to your caregiving responsibilities?	159	21
Do you feel that your team is flexible when it comes to your childcare responsibilities (e.g., when you've had to leave early or on time)?	158	22
Do you feel that your daily work life is well balanced with your caregiving responsibilities?	161	19

Have you ever had to listen to a comment about your family planning that you felt was inappropriate?	231	42
How often?	119	154
Have you ever been asked about your family plans during a job interview?	234	39

Questions that could only be answered meaningfully after a previous question had been answered with a specific response are shown in the bold-type boxes; the number of participants who answered the required question is indicated.

## 3 Results

### 3.1 Baseline parameters

In total, 271 responses were collected, of which 234 were fully completed. Incomplete questionnaires were also included in the analysis. The number of missing data points is listed in Table 1. Among all participants 173 (63.8%) were male and 98 (36.2%) female. The mean age was 43.7 years. Forty-four (16.2%) respondents were clinic directors or deputy clinic directors, 103 (38.0%) were attendings or section heads, and 100 (36.9%) were residents or board-certified neurosurgeons. Most respondents (83%, n = 225) worked full-time and 198 (73%) reported a workload exceeding 50 h per week. A total of 87.6% (n = 220) stated that they were in a committed relationship. Among all participants, 53% reported that their partners also worked in healthcare. One hundred and ninety-five (72.0%) of all participants had children. Childcare was mostly supported by kindergarten/daycare (49%) and family members (42%). Of all participants with children, most had two children (36%), followed by three children (30%) and one child (28.1%). Fewer than 6% of respondents with children had four or more children.

### 3.2 General career wise disadvantages due to caring

On a satisfaction scale from 0 to 100 (0 = negative, 100 = positive), the disadvantages experienced due to childcare were rated with a mean score of 48.1, while actual working conditions were rated at 31.4. In contrast, team flexibility (61.2) and management flexibility (52.4) received rather positive ratings. Fig. 1 illustrates the categorization of perceived disadvantages across these aspects using a color-coded scale, ranging from significant disadvantages (red, 0) on the left to no disadvantages (green, 100) on the right. Experience of disadvantage included being asked about family planning during job interviews (29%) and inappropriate remarks about family planning (50%). Among respondents younger than 30 years, 22% had been asked about family planning during a job interview. The corresponding proportions were 35% among those aged 31–40 years, 28% among those aged 41–50 years, and 22% among those aged 51–60 years and those older than 60 years. This age-related difference was statistically significant (p = 0.006). A reduction in working hours was perceived as beneficial for family life by 81% of respondents but was associated with concerns about career disadvantages in 64%. Overall, 72% of participants stated that a reduction in working hours led to conflicts within their team.

**Fig. 1:**
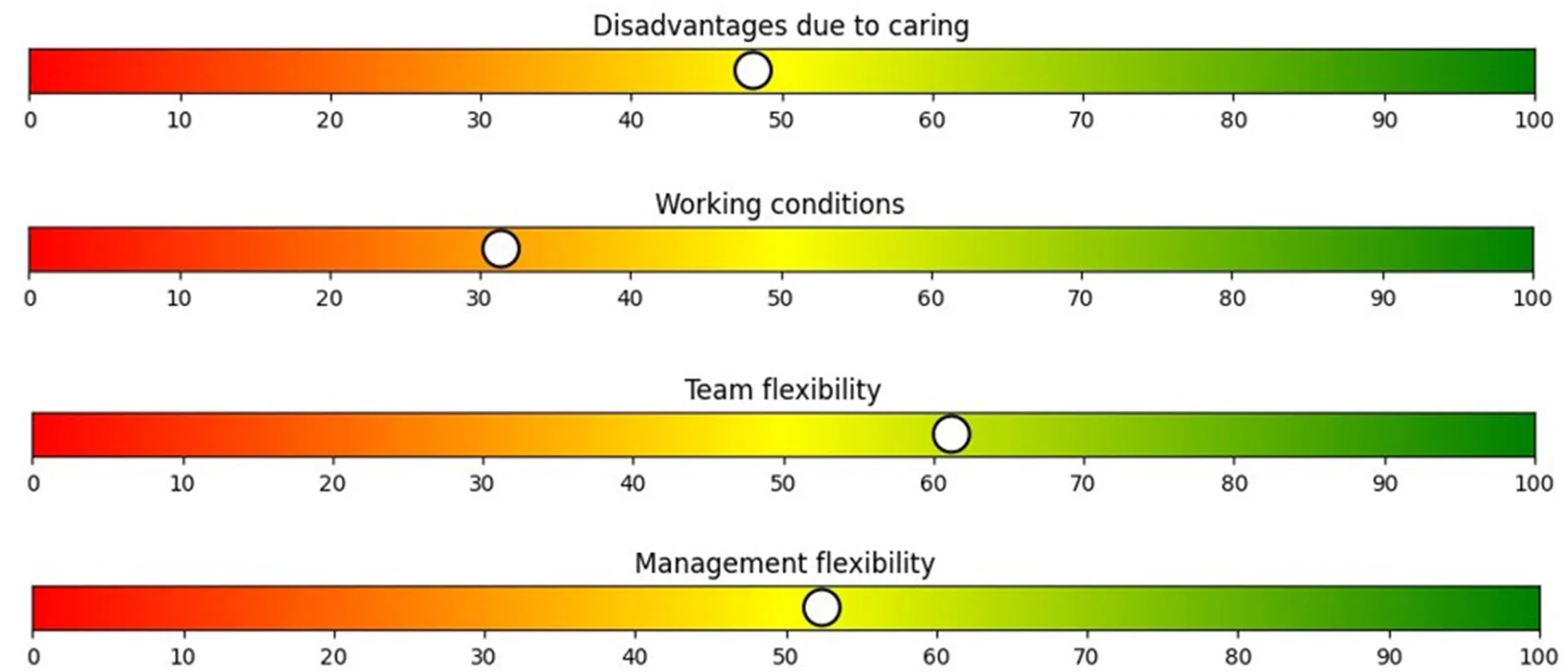
Perception of disadvantages related to childcare and working conditions.

### 3.3 Impact of part-time work

Among all participants currently working part-time (17%), male neurosurgeons reported significantly more often (p = 0.015) that they felt disadvantaged in their job by caring responsibilities. The distribution of part-time work by gender is shown in Fig. 2. On a scale from 0 (major disadvantage) to 100 (no disadvantage), the mean score was 77.4 for female neurosurgeons and 43.5 for male neurosurgeons. There were no significant differences between men and women in their perceptions of their supervisors' (p = 0.770) or colleagues’ (p = 0.490) flexibility with regard to caregiving responsibilities. Furthermore, there was no significant difference between genders when it came to assessing whether everyday working life was well suited to childcare (p = 0.543). Among participants planning to reduce their working hours in the future, there were no significant differences between male and female neurosurgeons in their assessments of team flexibility (p = 0.844) or employer flexibility (p = 0.565).

**Fig. 2:**
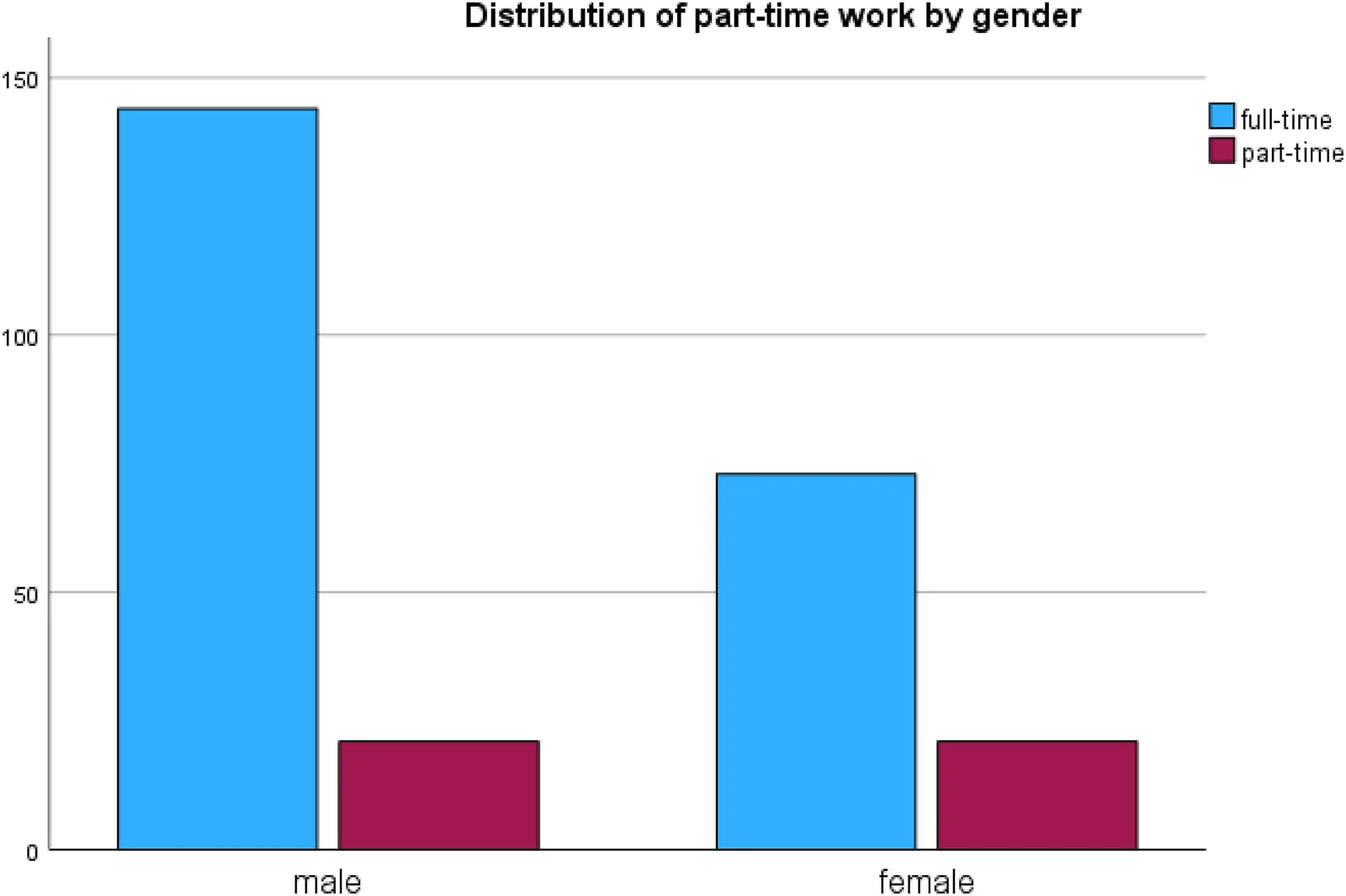
Distribution of part-time work by gender.

### 3.4 Gender disparities

Gender disparities are shown in Table 2, Table 3, Table 4. Among all participants 173 (63.8%) were male and 98 (36.2%) female. Female neurosurgeons were significantly younger than male colleagues (mean 38.8 vs. 46.3 years, p < 0.001) and were significantly less likely to hold leading positions (12.4% of women vs. 41.8% of men, p < 0.001). Aspects of private life are listed in Table 3: Overall, 78.3% of female and 93.0% of male neurosurgeons stated, that they were in a committed relationship (p < 0.001). Significantly more male (78%) than female neurosurgeons (61%) reported having children (p < 0.004). Nonetheless, 22.3% of women and only 12.7% of men worked part-time (p < 0.044) in this cohort. Questions and comments regarding family planning in the workplace are listed in Table 4: Female neurosurgeons encountered inappropriate comments on family planning significantly more often than their male colleagues (57.0% of women vs. 39.4% of men, p < 0.001) and were asked about family plans during job interviews significantly more often (40.9% of women vs. 21.4% of men, p < 0.001). The question of family planning in job interviews was significantly dependent on age for men (p = 0.011), with an increase in the frequency of the question among younger men. While 25% of men under the age of 30 and 24% of men between 31 and 40 had already been asked about their family planning, the figure was only 17% for those aged 41 to 50. Among women, there was no significant correlation between age and the question of family planning in job interviews (p = 0.312). However, the frequency of being asked about family planning decreased with age among women: 50% of those aged 41–50 years and 48% of those aged 31–40 years reported being asked this question, compared with only 20% of those younger than 30 years. Fig. 3 illustrates how the question of family planning in job interviews varies by age. Perceptions of disadvantages and the flexibility of supervisors and team members regarding childcare responsibilities were assessed on a continuous scale from 0 to 100. As the Shapiro–Wilk test indicated that the data were not normally distributed, a Mann–Whitney *U* test was applied. Regarding the perception of disadvantages related to childcare in a subgroup of all neurosurgeons with children, measured on a continuous scale from 0 (maximum perceived disadvantage) to 100 (no perceived disadvantage), the mean score was 42 for men and 65 for women (p < 0.001, Fig. 4). Regarding perceived supervisor flexibility in relation to childcare responsibilities (measured from 0 - “not flexible at all” to 100 - “completely flexible”), the mean score was 50 for men and 55 for women without statistically significant difference between genders (p = 0.506). Regarding perceived team flexibility in relation to childcare responsibilities (measured from 0 - “not flexible at all” to 100 - “completely flexible”), the mean score was 61 for men and 62 for women without statistically significance difference between genders (p = 0.826). Regarding the alignment of daily professional routines with childcare responsibilities, measured on a continuous scale from 0 (not well aligned) to 100 (perfectly aligned), the mean score was 29 for men and 37 for women, showing no statistically significant difference between genders (p = 0.355).

**Table 2:** Gender differences in the workplace.

Questions	Male N = 173	Female N = 98	p-value
**How old are you?**	Mean 46.3 years	Mean 38.8 years	p < 0.001
**Are you board certified?**			
Yes	132	63	p = 0.034
No	41	35	
**What is your postition in the hospital?**			
Chief physician	35	3	p<.001
Deputy chief physician	4	2	
Senior/managing physician	27	6	
Attending	45	21	
Section head	1	3	
Specialist	10	23	
Resident	36	31	
**How many teaching hours per week per semester do you hold?**			
<1	68	53	p = 0.044
1 – 2	61	23	
>2	35	13	
**Do you work full-time?**			
Yes	144	73	p = 0.044
No	21	21	
**How do you estimate your actual working hours per week?**			
10 – 19	3	0	p < 0.001
20 – 29	0	2	
30 – 39	2	14	
40 – 49	28	19	
> 50	123	57	

Univariate analysis - Significant differences between women and men working in neurosurgery.

**Table 3:** Gender differences in the private and family context.

Questions	Male N = 173	Female N = 98	p-value
**Are you in a committed relationship?**			
Yes	147	72	p < 0.001
No	11	20	
**Does your partner work full-time or part-time?**			
Full-time	55	61	p < 0.001
Part-time	74	7	
Other	20	0	
**Does your partner work in healthcare?**			
Yes	94	23	p < 0.001
No	55	49	
**Do you have children?**			
Yes	123	56	p = 0.004
No	35	36	

Univariate analysis - Significant differences between women and men working in neurosurgery.

**Table 4:** Gender differences in comments about family planning in the workplace.

Questions	Male N = 173	Female N = 98	p-value
**Have you ever had to listen to an inappropriate comment about your family planning?**			
Yes	56	59	p < 0.001
No	86	29	
**Have you ever been asked about your family plans during a job interview?**			
Yes	31	36	p < 0.001
No	114	52	
**Question about family planning in a job interview based on age (years), answer = yes**			
< 31	25%	20%	p = 0.006
31 – 40	24%	48%	
41 – 50	17%	50%	
51 – 60	20%	29%	
> 60	25%	0%	

Univariate analysis - Significant differences between women and men working in neurosurgery.

**Fig. 3:**
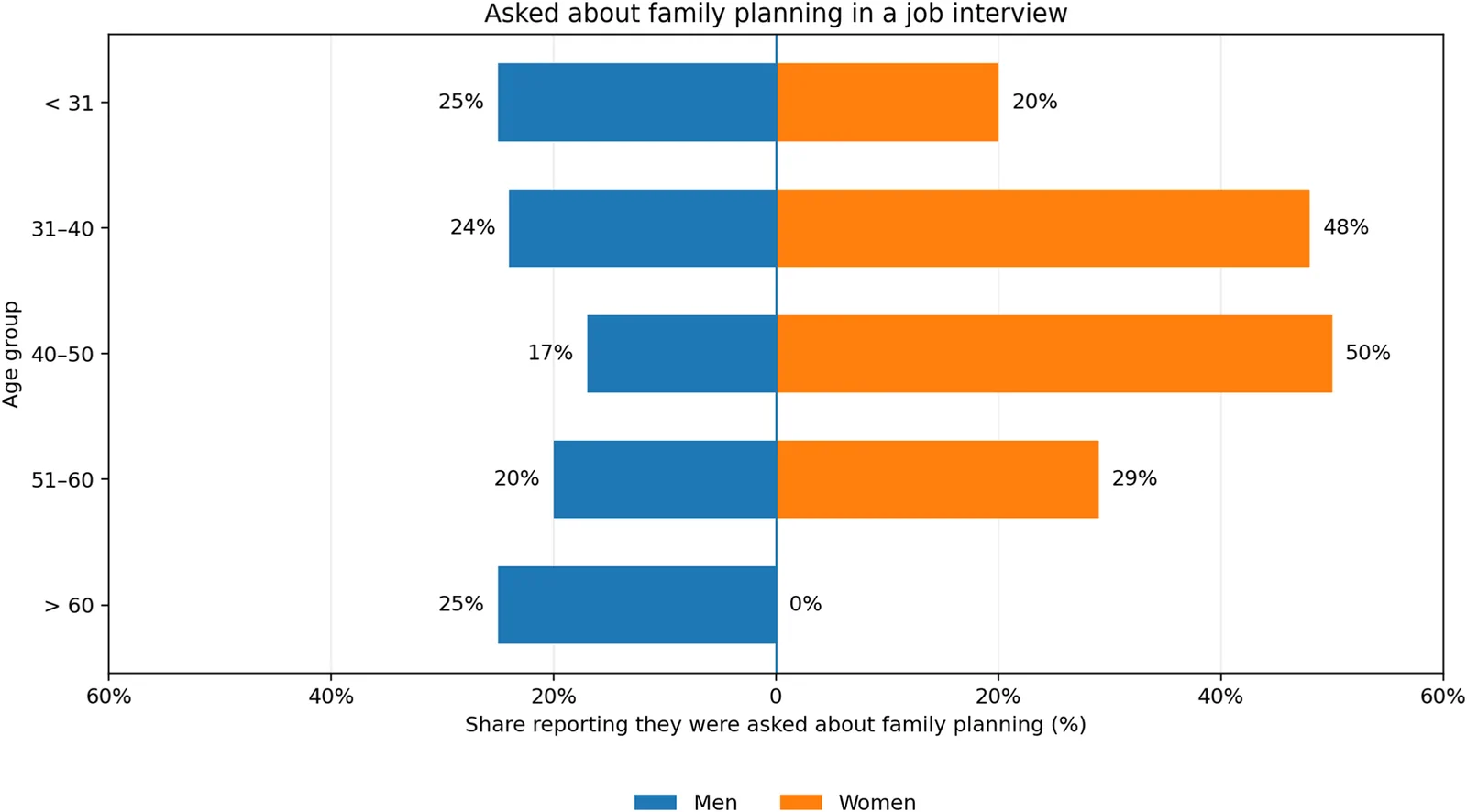
Age and gender distribution regarding questions about family planning in job interviews.

**Fig. 4:**
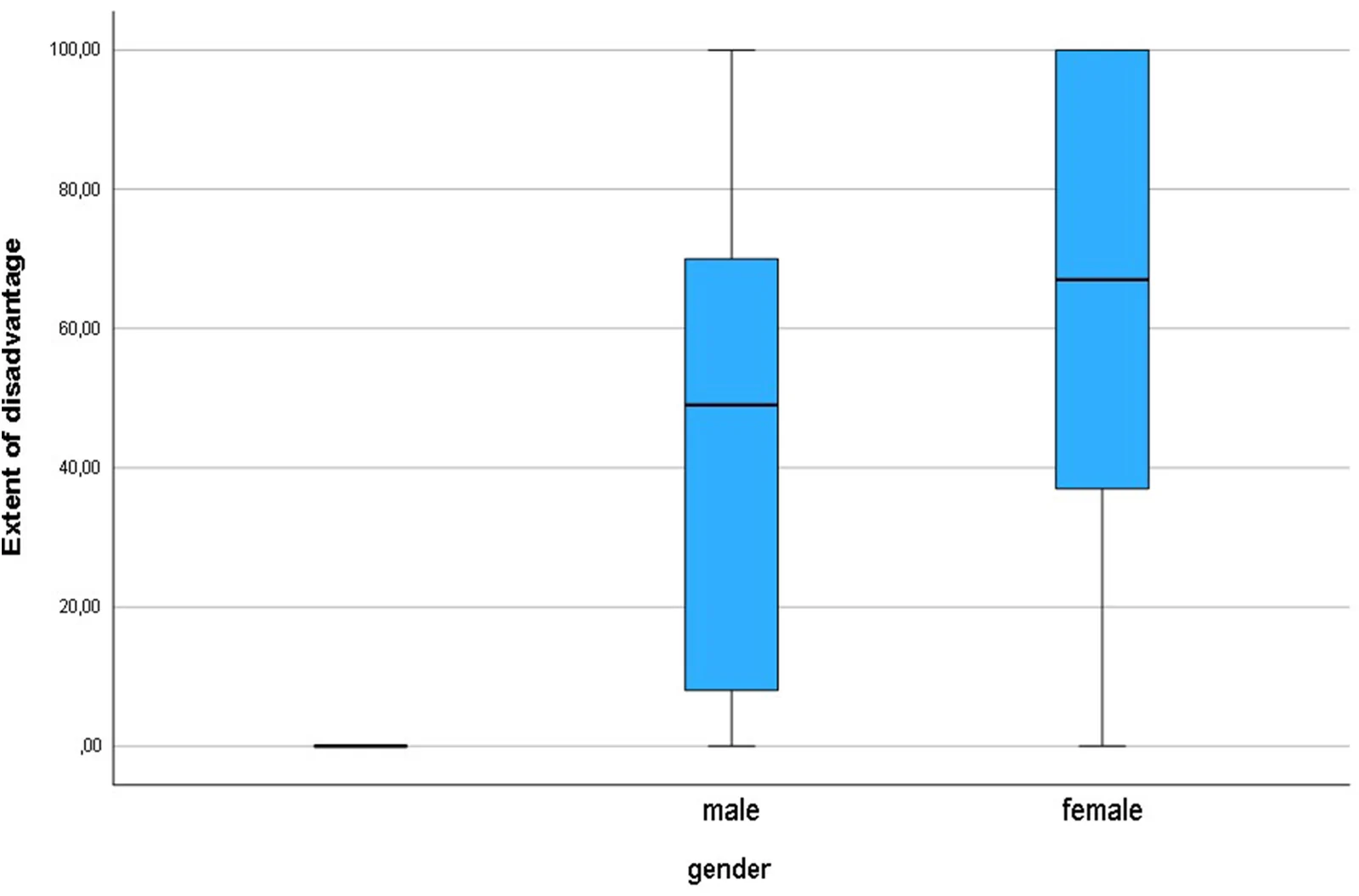
Perception of disadvantages related to childcare by gender.

## 4 Discussion

### 4.1 Principal findings

This study demonstrates that caregiving, parenthood, and professional life strongly influence each other among neurosurgeons in Germany. Although female neurosurgeons in our study are more often subject to discrimination related to family planning, male neurosurgeons also experience disadvantages when attempting to become involved in caregiving responsibilities. These findings underline that care-related disadvantages represent a broader issue beyond gender differences and require a comprehensive approach by future medical leaders.

### 4.2 Workplace conditions

Dissatisfaction with working conditions related to caregiving is best reflected in our scales measuring perceived disadvantages (0 = significant disadvantages, 100 = no disadvantages). In our study, satisfaction with workplace conditions that support childcare were rated low and fell within the negative range of the rating scale (score: 32.4). In contrast, the flexibility of the team (score: 61.2) and that of supervisors (score: 52.4) were both rated at least in the positive part of the rating scale. Nevertheless, 72% of participants answering this question in our survey stated that a reduction in working hours would lead to conflicts within their team. However, this statement must be interpreted with caution, as only 73 of the 273 participants responded to it. While both statements may seem contradictory at first glance, they may in fact reflect the same problem. The conditions at work that require teams to cover the absence of a pregnant colleague or a colleague on parental leave may contribute to the anticipated conflicts, despite the positive rating of team flexibility. This might promote discrimination against employees who want to have children, disadvantaging both men and women. The lowest rating for the workplace conditions in our study suggests that earlier studies focusing on disadvantages for women in surgery and neurosurgery alone might have overlooked a broader, more general problem that our study identifies as a key finding for the first time: the working conditions faced by physicians in the German healthcare system. Other, studies not specific to neurosurgery have already identified balancing work and family as a major challenge in the German working environment (Fuss et al., 2008). Recent studies have highlighted higher employee satisfaction with work–life balance in Scandinavian countries, particularly Norway (Rosta and Aasland, 2011; Voltmer et al., 2024). The team's willingness to be considerate of colleagues who are involved in childcare shows that the trend in our profession is toward mutual support, but this must be supported by appropriate structural conditions. These conditions have been unsatisfactory so far as mentioned above. Our data, which indicate dissatisfaction with working conditions but also suggest relatively greater flexibility among team members and supervisors, suggest that our work environment would, in principle, be open to change; however, rigid workplace structures have so far prevented this potential for change from being fully realized. As a result, current working conditions not only make it difficult to balance family and work but also contribute to discrimination against parents and the perpetuation of stereotypical gender roles.

### 4.3 Discrimination and gender roles

Men are significantly overrepresented in our cohort (63.8% vs. 36.2%), which is likely due to the large proportion of male neurosurgeons in Germany. The distribution of genders and positions by gender in our survey roughly corresponds to the distribution in Germany (Forster et al., 2022). However, the participation of male neurosurgeons in our survey also highlights the relevance of childcare-related issues to this group. Previous studies on the compatibility of family and professional life in surgery have mainly focused on the difficulties experienced by women (Wolfert et al., 2019; Casilla et al., 2022). For example, a survey showed that the pregnancies of female doctors are viewed negatively by their male colleagues (Casilla et al., 2022). In our study, significantly more men had children than women. Other studies have also shown that female surgeons had fewer children than their male colleagues (Au et al., 2021). On the one hand, this shows that balancing work and family life affects a large proportion of men, but on the other hand, it also suggests that women in neurosurgery may be more likely to postpone starting a family or decide against having children than men. This trend is likely influenced by the fact that female neurosurgeons were significantly younger than their male counterparts (38.8 vs. 46.3 years, p < 0.001). Another study has shown that residents postpone family planning due to their careers, which affects both genders (Reid et al., 2021). Although women had significantly fewer children than men, they were significantly more likely to be asked about family planning during job interviews, suggesting ongoing gender bias in recruitment practices. However, this finding should be interpreted with some caution, as some of these experiences may date back more than a decade, given the participants' ages. Notably, women aged 31–50 years reported being asked about family planning particularly frequently (48–50%), whereas the proportion was lower among women under 30 years (20%). Among men, the trend was slightly reversed, with 17% of those aged 41–50 years and 24–25% of those under 41 years reporting such questions. The small number of women over 50 years of age in neurosurgery should also be considered a limitation.

Regarding the perception of disadvantages related to childcare, measured on a continuous scale from 0 (maximum perceived disadvantage) to 100 (no perceived disadvantage), the mean score was 42 for men and 61 for women (p < 0.0001), which means that men perceived disadvantages related to childcare as more relevant. This is an interesting fact because, in our study, men were significantly more likely to hold leadership positions and significantly less likely to be subjected to inappropriate comments about family planning from colleagues or during job interviews. Our questionnaire cannot fully identify the cause of this perceived disadvantage, which is felt more frequently among men. Neither the flexibility of the employer nor that of the team, nor the general working conditions, were perceived as significantly worse by men than by women. The proportion of men working part-time was also lower. A German study showed that men experience higher levels of parental stress regardless of whether they take parental leave (Krüger and Rüther, 2022). Nonetheless, men across Germany still take significantly less parental leave than women, but the proportion of men has doubled in the last 10 years ((Destatis and) SB, 2024). This trend may contribute to the finding that younger men in our cohort were more frequently asked about family planning during job interviews. Taken together, these findings suggest that men, as they become more involved in caring for their children, may fear career-related consequences similar to those experienced by women. A study interviewing fathers found that they feared that taking parental leave might negatively affect their promotion prospects, their current positions, or their ability to compete with their colleagues (Mita et al., 2024). Ultimately, the parameters we collected do not allow us to clearly identify why men feel so strongly disadvantaged, and further studies are needed, which, in addition to psychological aspects, also examine factors such as loss of employment or poorer surgical training due to child-caring responsibilities. A larger sample size will likely be necessary to investigate this further. A psychological study showed that fathers are more likely to take parental leave if they receive support from their partner and that working conditions play a less significant role (Scheifele et al., 2024). Furthermore, expected negative reactions played a key role as well (Scheifele et al., 2024). This suggests that, in addition to the working conditions, older gender models also play a role in making it difficult for male and female neurosurgeons to balance family and their career. The phenomenon, in which one experiences social punishment for adopting characteristics of the other role, is known as the backlash effect (Rudman and Fairchild, 2004). Further psychological studies have shown that masculinity, in particular, is perceived as fragile and easily threatened (Bosson and Vandello, 2011; Vandello et al., 2008). Therefore, continued attention to gender role perceptions in neurosurgery is essential to achieve genuine equality in both professional and caregiving contexts. Institutional measures—such as flexible working models, structured parental leave policies for both parents, accessible childcare, and a workplace culture that actively supports fathers in taking an equal role in childcare are crucial to reduce disadvantages and promote equity in neurosurgery. Such measures may even help prevent doctors from moving abroad or switching to non-clinical occupations (Fuss et al., 2008). The unequal distribution of childcare might have a negative impact on both genders. Partners of men who take on less childcare may face a greater burden, while men who are not encouraged to become more involved in childcare due to inadequate workplace conditions may miss out on valuable time with their families. Furthermore, there is evidence that involvement in childcare can have positive effects on men's mental health. (Knoester et al., 2007).

### 4.4 Limitations

Although we consider our participant numbers to be comparatively high, they may not reflect the experience of all neurosurgeons in Germany. Compared with the actual distribution, residents are significantly underrepresented in our cohort. Additionally, the different age distributions between men and women may have introduced bias. The questionnaire was not subjected to formal psychometric validation. Furthermore, the absence of institutional information, which was deliberately omitted to reduce the risk of re-identification, prevented us from examining potential differences between institutional settings and limits the generalizability of the findings across these settings. In addition, a substantial proportion of participants did not respond to two questions concerning the anticipated negative consequences of a future reduction in working hours, either for their team or for themselves if the reduction were mandated by their supervisor. Partially missing data limits the interpretation of these findings.

## 5 Conclusion

Our study provides a novel perspective on the compatibility of caregiving and professional life in neurosurgery by highlighting that caregiving-related disadvantages are not limited to women but also affect male neurosurgeons. Still, women continue to experience these disadvantages to a greater extent. Notably, younger men appear to face challenges related to caregiving that resemble those traditionally reported by women. Despite being less likely to work part-time, men perceived childcare-related career disadvantages as particularly significant. Across both genders, workplace conditions emerged as the most problematic aspect of balancing professional responsibilities and caregiving, suggesting that structural barriers may extend beyond gender-specific discrimination. The factors underlying these perceived disadvantages remain incompletely understood and warrant further investigation. However, the relatively positive assessments of team and supervisor flexibility suggest that there may be considerable potential for improvement if individual willingness to accommodate caregiving responsibilities is supported by structural changes. By bringing the experiences of male neurosurgeons into focus, our findings broaden the discussion of gender equality in neurosurgery and underscore the need for working conditions that enable both women and men to participate in caregiving without compromising their careers.

## Declaration of generative AI and AI-assisted technologies in the manuscript preparation process

During the preparation of this work the authors used DeepL in order to improve english language. After using this service, the authors reviewed and edited the content as needed and take full responsibility for the content of the published article.

## Funding

This research did not receive any specific grant from funding agencies in the public, commercial, or not-for-profit sectors.

## Conflict of interest

The authors declare no conflict of interest.
