# Supplementary material for: Family and neurosurgery - a balancing act between vocation and responsibility
Source: Brain Spine. 2026 Oct 23;6:107489. doi: 10.1016/j.bas.2026.107489 (PMC13721009; doi:10.1016/j.bas.2026.107489)
Supplement: Supplementary file 1 [file mmc1.pdf]

Familie und Neurochirurgie - Ein Balanceakt zwischen Berufung und Verantwortung

1. Mit der Teilnahme dieser Umfrage erkläre ich mich einverstanden, dass meine Daten anonymisiert verwendet und publiziert werden dürfen und dass ich anschließend von der Teilnahme nicht zurücktreten kann.

- Ja
- Nein -> Ende der Befragung

2. Welche Geschlechtsidentität haben Sie?

- Männlich
- Weiblich
- Divers
- Keine der oben genannten

3. Wie alt sind Sie?

- Freitext

4. Haben Sie bereits einen Facharzttitel?

- Ja
- Nein

5. In welchem Weiterbildungsjahr befinden Sie sich?

- Freitexteingabe

6. Was ist Ihre Position innerhalb der Klinik?

- Chefarzt, Chefärztin/ KlinikdirektorIn
- Stellv. KlinikleiterIn, Leitender Oberarzt, Leitende Oberärztin/ Geschäftsführe/r Oberarzt, Oberärztin
- Oberarzt, Oberärztin
- Facharzt, Fachärztin
- SektionsleiterIn

- Assistenzarzt, Assistenzärztin
- Keine der oben genannten

7. Wie viele Semesterwochenstunden sind Sie in der Lehre tätig?

- <1
- 1-2
- >2

8. Haben Sie weitere Zusatzaufgaben innerhalb der Klinik (z.B. Hygienebeauftragte/r oder Datenschutzbeauftragte/r)?

- Ja
- Nein

9. Arbeiten Sie aktuell in Vollzeit?

- Ja
- Nein

10. Haben Sie vor Ihre Arbeitszeit in Zukunft zu reduzieren?

- Ja
- Nein

11. Haben Sie das Gefühl, dass eine zukünftige Arbeitszeitreduktion in Ihrem Team mit Nachteilen verbunden wäre?

- Ja
- Nein

12. Haben Sie das Gefühl, dass eine Arbeitszeitreduktion durch Ihre/n Vorgesetzte/n einen Nachteil für Sie bedeuten würde?

- Ja
- Nein

13. Haben Sie schon einmal zuvor die Arbeitszeit reduziert?

- Ja
- Nein

14. Auf wie viel Prozent der ursprünglichen Wochenstunden haben Sie reduziert?

- 10-90 jeweils

15. Hatten sie durch die Arbeitszeitreduktion eine Erleichterung im Privatleben?

- Ja
- Nein

16. Was ist Ihre aktuelle wöchentliche Arbeitzeit laut Arbeitsvertrag ohne *opt out*?

- Freitext

17. Wie schätzen Sie ihre tatsächliche Arbeitszeit pro Woche in Stunden ein?
- 10-19
- 20-29
- 30-39
- 40-49
- >50

18. Leben Sie in einer festen Partnerschaft?

- Ja
- Nein

19. Ist Ihr/e PartnerIn erwerbstätig?

- Ja
- Nein

20. Arbeitet Ihr/e PartnerIn in Vollzeit oder Teilzeit?

- Vollzeit
- Teilzeit
- Keine der oben genannten

21. Arbeitet Ihr/e PartnerIn im Gesundheitswesen?

- Ja
- Nein

22. Haben Sie Kinder.

- Ja
- Nein

23. Wie viele Kinder haben Sie.
- 1->10

24. Wie sind die Kinder versorgt?

- Ausschließlich durch mich - Durch beide Partner gleichermaßen- Ausschließlich durch meinen Partner (Schieberegler)

25. Wie sind die Kinder versorgt?

- Großeltern und Familie
- Nanny/Babysitting
- AuPair
- Tagesmutter/-eltern
- Kindergarten/ Krippe
- Private Elterninitiative
- Keine der oben genannten

26. Haben Sie schon einmal Benachteiligung aufgrund der Caring-Aufgaben empfunden?

    - (Schieberegler)

27. Wie häufig.

    - (Schieberegler)

28. Haben Sie das Gefühl, dass Ihr/e Vorgesetzte/r flexibel bezüglich Ihrer Caring-Aufgaben ist?

- (Schieberegler)

29. Haben Sie das Gefühl, dass ihr Team flexibel bezüglich Ihrer Caring(Kinderbetreuungs-)Aufgaben ist (z. B. Wenn Sie einmal früher oder pünktlich gehen mussten)?

- (Schieberegler)

30. Haben Sie das Gefühl, dass ihr Berufsalltag gut auf Ihre Caring -Aufgaben abgestimmt ist?

- (Schieberegler)

31. Haben Sie sich schon einmal eine Ihrer Meinung nach unangebrachte Aussage bezüglich Ihrer Familienplanung anhören müssen?

- Ja
- Nein

32. Wie häufig?

- (Schieberegler)

33. Wurden Sie schon einmal in einem Bewerbungsgespräch nach Ihrer Familienplanung gefragt?

- Nein
- Ja
